# Automated Breast Volume Scanner (ABVS)-Based Radiomic Nomogram: A Potential Tool for Reducing Unnecessary Biopsies of BI-RADS 4 Lesions

**DOI:** 10.3390/diagnostics12010172

**Published:** 2022-01-12

**Authors:** Shi-Jie Wang, Hua-Qing Liu, Tao Yang, Ming-Quan Huang, Bo-Wen Zheng, Tao Wu, Chen Qiu, Lan-Qing Han, Jie Ren

**Affiliations:** 1 Department of Medical Ultrasonics, The Third Affiliated Hospital of Sun Yat-sen University, Guangzhou 510630, China; wangshj37@mail2.sysu.edu.cn (S.-J.W.); bovenzu@126.com (B.-W.Z.); wutao22@mail.sysu.edu.cn (T.W.); qiuch3@mail.sysu.edu.cn (C.Q.); 2 Artificial Intelligence Innovation Center, Research Institute of Tsinghua, Pearl River Delta, Guangzhou 510630, China; liuhq@tsinghua-gd.org (H.-Q.L.); hanlance@tsinghua-gd.org (L.-Q.H.); 3 Department of Ultrasound, The Affiliated Hospital of Southwest Medical University, Luzhou 646099, China; jj080212@126.com; 4 Department of Breast Surgery, The Affiliated Hospital of Southwest Medical University, Luzhou 646099, China; huangmingquan2022@163.com

**Keywords:** radiomics, nomogram, automated breast volume scanner, breast neoplasms

## Abstract

Improving the assessment of breast imaging reporting and data system (BI-RADS) 4 lesions and reducing unnecessary biopsies are urgent clinical issues. In this prospective study, a radiomic nomogram based on the automated breast volume scanner (ABVS) was constructed to identify benign and malignant BI-RADS 4 lesions and evaluate its value in reducing unnecessary biopsies. A total of 223 histologically confirmed BI-RADS 4 lesions were enrolled and assigned to the training and validation cohorts. A radiomic score was generated from the axial, sagittal, and coronal ABVS images. Combining the radiomic score and clinical-ultrasound factors, a radiomic nomogram was developed by multivariate logistic regression analysis. The nomogram integrating the radiomic score, lesion size, and BI-RADS 4 subcategories showed good discrimination between malignant and benign BI-RADS 4 lesions in the training (AUC, 0.959) and validation (AUC, 0.925) cohorts. Moreover, 42.5% of unnecessary biopsies would be reduced by using the nomogram, but nine (4%) malignant BI-RADS 4 lesions were unfortunately missed, of which 4A (77.8%) and small-sized (<10 mm) lesions (66.7%) accounted for the majority. The ABVS radiomics nomogram may be a potential tool to reduce unnecessary biopsies of BI-RADS 4 lesions, but its ability to detect small BI-RADS 4A lesions needs to be improved.

## 1. Introduction

Breast cancer is still the most common malignant tumor and the leading cause of cancer-related death in females [1]. Early diagnosis and treatment of breast cancer can significantly improve the survival rate and quality of life [2]. Conventional ultrasound (US) plays an important role in breast cancer screening and the differential diagnosis of breast lesions [3]. Breast lesions detected by US can be classified into seven categories (categories 0–6) according to the fifth edition of the Breast Imaging Reporting and Data System (BI-RADS) [4]. Among them, BI-RADS US category 4 (hereinafter referred to as BI-RADS 4) represents possibly malignant lesions, and biopsy is recommended [5,6]. However, approximately 67–78% of biopsies are unnecessary for BI-RADS 4 lesions due to the broad-range malignant potential (3% to 94%) [7,8,9]. Moreover, unnecessary biopsies are associated with negative consequences such as pain, anxiety, direct financial burden, and related complications [10]. Therefore, in the era of precision medicine, improving the assessment of BI-RADS 4 lesions and reducing unnecessary biopsies are clinical problems that need to be solved.

Recently, a noninvasive, quantitative and objective image analysis method named radiomic nomogram has attracted attention. It can extract high-throughput quantitative features that may not be observed directly by the naked eye from single or multiple medical images, and subsequently combine these features with clinical information to improve disease diagnosis and prognostic evaluation [11]. A large number of studies have reported the application of radiomic nomograms based on various imaging modalities, such as computed tomography (CT), magnetic resonance imaging (MRI), positron emission tomography-computed tomography (PET-CT), and US, which have showed great potential in the classification and prediction of breast cancer [12,13,14,15,16]. However, only a few studies have focused on BI-RADS 4 lesions [15,16], and no studies have been based on automatic breast volume scanner (ABVS).

As an emerging US technology, the ABVS automatically scans the breast based on a special high-frequency broadband transducer and obtains standardized, repeatable, and high-resolution US images [17]. In 2012, the Food and Drug Administration approved the use of ABVS for breast screening, especially for asymptomatic women with dense breasts [18], and some unique features of ABVS may provide additional information for distinguishing benign and malignant breast lesions [19,20]. Specifically, the retraction phenomenon on the coronal plane of ABVS presents as a stellate pattern around the lesion, which has high sensitivity (80–89%) and specificity (96–100%) for breast cancer [20,21,22]. Furthermore, the standardized, reproducible and high-resolution characteristics of ABVS images will be more suitable for radiomics analysis, according to the radiomics quality score (RQS) proposed by Lambin et al. [23]. However, to our knowledge, whether ABVS-based radiomic nomogram has potential to identify benign and malignant breast lesions, especially BI-RADS 4 lesions, remains unknown.

Therefore, for bridging the gap and taking full use of ABVS images to promote precision medicine, this prospective study aimed to investigate the ability of the ABVS radiomic nomogram to distinguish benign and malignant BI-RADS 4 lesions, and evaluate its potential value in reducing unnecessary biopsies of these lesions.

## 2. Materials and Methods

### 2.1. Patient Selection

This study was approved by the Institutional Review Board of our hospital (KY2020163), and written informed consent was obtained from all patients. Between April and August 2020, consecutive women with US-detected BI-RADS 4 lesions and scheduled for US-guided core needle biopsy were invited to participate in the study. The inclusion criteria were as follows: (a) each BI-RADS 4 lesion was confirmed by a senior radiologist (with seven years of breast US experience), and ultimately assigned a subcategory (4A, 4B, or 4C) according to the second edition of the ACR BI-RADS US atlas; (b) subjects aged 18–80 years; and (c) subjects who voluntarily agreed to participate and signed informed consent forms. The exclusion criteria were as follows: (a) women who were not suitable for ABVS, such as pregnancy, breastfeeding, or breasts with implants; (b) women with previous breast surgery or percutaneous biopsy during the preceding 12 months; (c) BI-RADS 4 lesions with unclear ABVS images; and (d) absence of a definitive pathological diagnosis and incomplete clinical data. Finally, all BI-RADS 4 lesions included in this study were randomly divided into a training cohort and a validation cohort at a ratio of 8:2.

Information on menopausal status, oral contraceptives, smoking history, alcohol consumption history, and family history of breast or ovarian cancer was obtained directly from the patient. Breast density was assessed subjectively from digital mammography and classified as A, B, C, or D accordance with the American College of Radiology. The following US features were also recorded: lesion size, location of the lesion (left or right), shape (regular or irregular), orientation (parallel or not), posterior echo (enhancement, shadowing, combined pattern or no posterior echo), echo pattern (hypoechoic, hyperechoic or complex cystic and solid), and calcification (yes or no).

### 2.2. ABVS Examination

ABVS examination was performed by one of two well-trained radiologists using the ACUSON S2000 Automated Breast Volume Scanner (Siemens Medical Solutions, Inc., Mountain View, CA, USA). ABVS consists of a US scanner and a special stationary device with a linear array transducer (5–14 MHz bandwidth), which moves automatically in a scan box. A replaceable membrane covers the transducer to facilitate sufficient contact with the breast. Patients lay in the supine or lateral position with their arms above their head. The medical gel was placed evenly on the breast, and then the scan box was placed on the breast with appropriate compression to improve image quality. According to the size of the breast and the location of the target lesion, the appropriate scan depth was selected. The ABVS examination was continuous and automated, and the patient was asked to breathe gently and not to move during the examination. After the examination, axial ABVS images were sent to a dedicated workstation, and sagittal and coronal images were reconstructed automatically. Finally, the axial, sagittal, and coronal ABVS images showing the largest lesions were selected for further image segmentation and feature extraction.

### 2.3. Breast Biopsy

Within one week after ABVS examination, US-guided core-needle biopsy was performed by experienced US interventional doctors. According to the standard biopsy procedure, four to eight samples per lesion were acquired using an automatic biopsy gun with a 14G or 16G needle [24]. Breast pathologists with at least 10 years of experience performed histopathological analysis. The final pathological diagnoses were classified as either benign or malignant, in which malignancy was defined as infiltrating carcinoma or ductal carcinoma in situ, and all other diagnoses were considered benign. For lesions with high-risk diagnosis (atypical findings, complex sclerosis, or papillary lesions) obtained by percutaneous biopsy, the final diagnosis was based on the surgical pathology.

### 2.4. ABVS Radiomic Score

Image segmentation and radiomic feature extraction were performed using MaZda software (version 4.6, www.eletel.p.lodz.pl/programy/mazda/, accessed on 1 January 2022), which was originally used for automatic texture analysis of MRI, and now has been extended to the investigation of any kind of digital images [25]. ABVS images were normalized using Mazda’s built-in normalization method before segmentation to minimize the influence of contrast and brightness variation. The lesion was manually delineated on the axial, sagittal, and coronal ABVS images by one trained radiologist (R1, with seven years of breast US experience). Contouring was drawn within the border of the lesion, and the adjacent parenchyma and fat were carefully avoided. In total, seven common radiomics feature groups, such as histogram, geometry, absolute gradient, gray level cooccurrence matrix, run matrix, autoregressive model, and wavelet transform, were automatically extracted from MaZda (Supplementary Table S1). In addition, all radiomic features were rescaled via Z-score normalization to facilitate subsequent statistical analysis.

The reproducibility of ABVS radiomic features extraction was evaluated based on the intra-operator and inter-operator findings. A total of two weeks after extraction of radiomic features in the training cohort, R1 repeated the extraction of ABVS radiomic features to evaluate the intra-operator agreement. Another trained radiologist (R2, with four years of breast US experience) performed the same procedure in the training cohort for the evaluation inter-operator agreement on features extraction. The intra- and interclass correlation coefficients (ICCs) greater than 0.75 were considered good agreement.

A three-step feature selection technique was used to select important radiomic features. First, the ABVS radiomic features with ICCs less than 0.75 were eliminated. Second, the minimum redundancy maximum relevance (mRMR) algorithm was used to select the remaining features. Third, the least absolute shrinkage and selection operator (LASSO) logistic regression method with 10-fold cross validation was applied to select the key features from the training cohort. Then, the radiomic score was built through a linear combination of the selected features weighted by the respective coefficients.

### 2.5. ABVS Radiomic Nomogram and Clinical Significance

**Figure 1:**
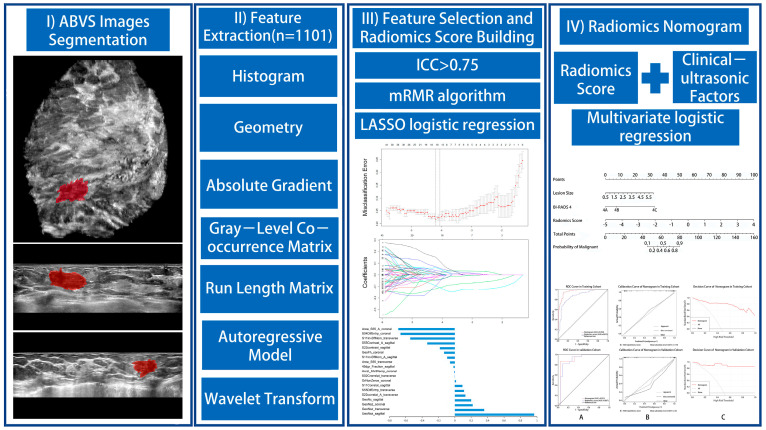
Workflow of necessary steps in this study. BI-RADS 4 lesions are manually segmented on axial, sagittal, and coronal ABVS images. Radiomic features are automatically extracted by MaZda software. A three-step feature selection technique to identify key radiomic features and incorporate them into the radiomic score was used. Combined with radiomic score and clinical-ultrasound factors, the ABVS radiomic nomogram was constructed by univariate and multivariate logistic regression analyses. The performance of the nomogram was assessed by the area under a receiver operating characteristic (ROC) curve, calibration curve, and decision curve analysis.

Univariate logistic regression and multivariate logistic regression analyses were performed to select the independent risk factors for malignant BI-RADS 4 lesions. The ABVS radiomic nomogram was developed based on the independent risk factors in the training cohort. The workflow of the study was shown in Figure 1. Calibration of the nomogram was assessed with a calibration curve, and the goodness-of-fit was evaluated with the Hosmer–Lemeshow test. The discrimination performance of the nomogram was evaluated using the area under the receiver operator characteristic (ROC) curve (AUC). Other discrimination metrics, including accuracy, sensitivity, specificity, positive predictive value (PPV), and negative predictive value (NPV), were also measured. In the validation cohort, the calibration and discrimination performances of the nomogram were validated, respectively.

Decision curve analysis (DCA) was performed to determine the clinical usefulness of the ABVS radiomic nomogram by quantifying the net benefits at different threshold probabilities in the training and validation cohorts. Furthermore, the malignant probability (defined as the Nomo-score in this study) of each BI-RADS 4 lesion was calculated according to the ABVS radiomic nomogram, and the optimal cutoff value was determined by the maximum Youden index. If biopsy was performed when the Nomo-score of the BI-RADS 4 lesion was greater than the cutoff value, the reduction in the unnecessary biopsy rate and the missed diagnosis rate of malignant lesions were calculated.

### 2.6. Statistical Analysis

Statistical analyses were performed using IBM SPSS Statistics 24 software and R software version 3.3.3. Continuous variables are expressed as the mean and standard deviation (SD), whereas categorical variables are expressed as the frequency and proportion. The chi-squared test or Fisher’s exact test was used to compare categorical variables. The Kolmogorov–Smirnov test was used to check whether the variables were normally distributed. Student’s *t*-test was used to compare continuous variables with a normal distribution; otherwise, the Mann–Whitney U-test was used. R software was used to develop and assess the radiomic score and the radiomic nomogram (see Supplemental S1). Statistical significance was accepted at *p* < 0.05.

## 3. Results

### 3.1. Clinical Characteristics

Between April and August 2020, 215 women were recruited and 22 women were subsequent exclusions: 11 retrieved consents (rejection of breast biopsy or ABVS examination); seven acquired unclear ABVS images due to different artifacts; and four had indefinite pathological diagnosis. Finally, 193 women (mean age, 49.4 ± 12.3 years; range 25 to 79 years) with 223 BI-RADS 4 lesions were enrolled in this study. The average size of the lesions was 19.5 ± 10.2 mm (range, 5–59 mm). The subcategories of BI-RADS 4 lesions were as follows: 104 lesions (46.6%) were BI-RADS 4A, 43 lesions (19.3%) were BI-RADS 4B, and 76 lesions (34.1%) were BI-RADS 4C. Histopathology analysis confirmed 103 of the 223 BI-RADS 4 lesions (46.2%) as malignant, and the malignancy rates of 4A, 4B and 4C were 11.5% (12/104), 44.2% (19/43) and 94.7% (72/76).

All 223 BI-RADS 4 lesions were randomly divided into a training cohort (*n* = 178) and a validation cohort (*n* = 45) in this study. The clinical characteristics and US features of the two cohorts are shown in Table 1. Between the two cohorts, there was no difference in the frequency of malignant lesions (81/178, 45.5% vs. 22/45, 48.9%, *p* = 0.684). In addition, significant differences in other clinical and US characteristics were also not observed in the training cohort and validation cohort (*p* > 0.05). This suggests that the training cohort and validation cohort were comparable in these characteristics. We also investigated the above basic information between the malignant and benign lesions in the training and validation cohorts (Supplementary Table S2).

### 3.2. ABVS Radiomic Score

**Figure 2:**
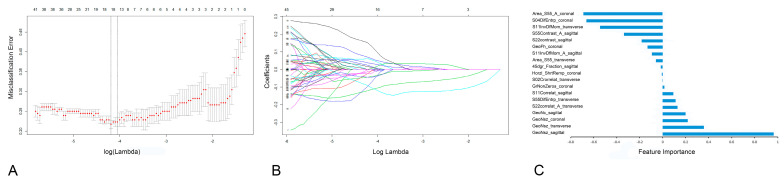
A total of 19 key radiomic features were identified by least absolute shrinkage and selection operator regression (LASSO). (**A**) Repeat 50 times of 10-fold cross-validation process to identification the optimal penalization coefficient lambda (λ) in the LASSO model, and 19 nonzero coefficients were obtained according to the λ. The red dots represent the mean value of the target parameters. (**B**) LASSO coefficient profiles were plotted against the log (λ) sequence. (**C**) Ranked the importance of 19 key features.

A total of 1101 features were obtained from axial, sagittal, and coronal ABVS images for each BI-RADS 4 lesion. The intra- and inter-operator reproducibility of ABVS radiomic features extraction were further assessed. The intra-class correlation coefficient of R1 in two extractions ranged from 0.815 to 0.982. The inter-class correlation coefficient of extraction by R1 and R2 ranged from 0.656 to 0.904. Then, 891 (80.9%) radiomic features with favorable intra- and inter-operator reproducibility (ICC > 0.75) were reserved for subsequent analysis. Of these features, 19 key features (six from coronal images of ABVS, six from axial images, and seven from sagittal images) were screened out with the mRMR algorithm and LASSO logistic regression in the training cohort (Figure 2). Based on the 19 features and the weighting by their respective coefficients, the radiomic score was established, and the formula is presented in Supplemental S2. The AUC of ABVS radiomic score was 0.875 (95% CI, 0.824–0.927) in the training cohort and 0.897 (95% CI, 0.835–0.936) in the validation cohort.

### 3.3. ABVS Radiomic Nomogram

**Figure 3:**
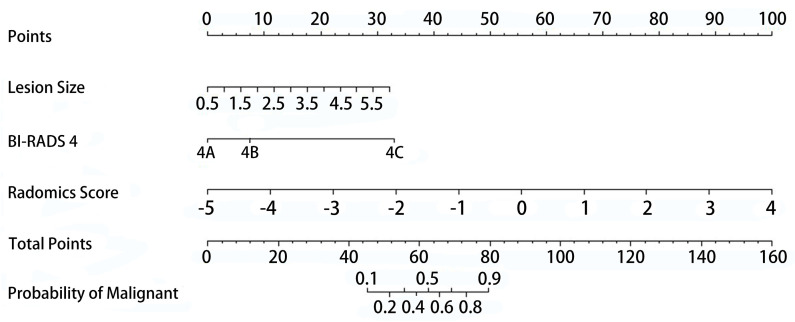
Radiomic nomogram. The radiomic nomogram was developed by combining the radiomic score, lesion size and BI-RADS 4 subcategory to distinguish benign and malignant BI-RADS 4 lesions.

**Figure 4:**
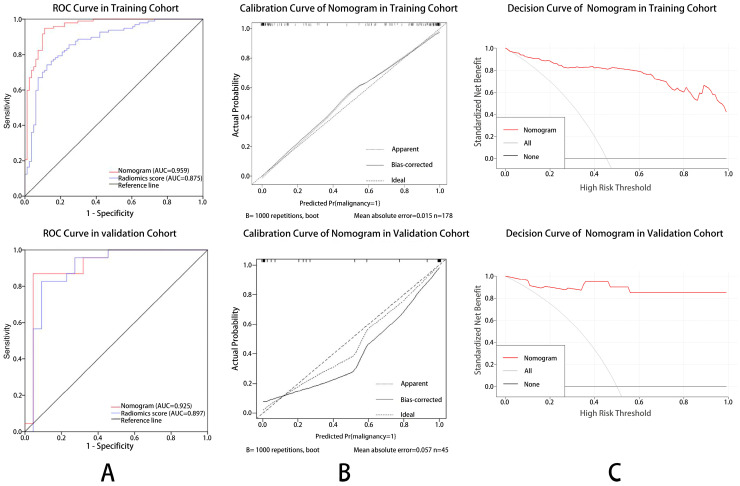
Receiver operating characteristic curves (ROC), calibration curves and decision curve analysis of the ABVS radiomic nomogram in training and validation cohort. (**A**) In the training and validation cohort, the AUC of the radiomic nomogram (red lines) were 0.959 and 0.925, and the AUC of the radiomic score (blue line) were 0.875 and 0.897, respectively. (**B**) The calibration curves of the nomogram were close to the standard curves in the training and validation cohort, which suggested that the nomogram was well-calibrated. (**C**) Decision curve showed that when the threshold probability was more than 5% in the training cohort and 2% in the validation cohort, using the nomogram had more benefit than either assuming all BI-RADS 4 lesions were malignant or assuming all lesions were benign.

Univariate and multivariate logistic regression analyses showed that the radiomic score (OR: 0.206, 95% CI: 0.104–0.407, *p* < 0.001), lesion size (OR: 2.838, 95% CI: 1.468–5.485, *p* = 0.002) and BI-RADS 4 subcategories (OR: 4.794, 95% CI: 0.803–8.624, *p* < 0.001) were identified as independent risk factors for malignant BI-RADS 4 lesions (Table 2). The ABVS radiomic nomogram was established by incorporating the above risk factors (Figure 3). Compared with the ABVS radiomic score, the performance of the ABVS radiomic nomogram was significantly improved in both the training cohort [AUC, 0.959 (95% CI, 0.933–0.984) vs. 0.875 (95% CI, 0.824–0.927); Z = 2.95, *p* = 0.002] and the validation cohort [AUC, 0.925 (95% CI, 0.824–0.971) vs. 0.897 (95% CI, 0.835–0.936); Z = 1.65, *p* = 0.04] (Figure 4A). The calibration curves of the ABVS radiomic nomogram were close to the standard curves in the training cohort and the validation cohort, which suggested that the nomogram was well calibrated (Figure 4B). The optimal cutoff value of the ABVS radiomic nomogram in this study was 0.4, and the accuracy, sensitivity, specificity, PPV, and NPV were 90.6%, 88.7%, 92.3%, 91.3%, and 90.0%, respectively. We also calculated the above performance indicators in the training and validation cohorts (Table 3).

### 3.4. Clinical Value of The ABVS Radiomic Nomogram

The decision curve analysis was used to assess the clinical significance of ABVS radiomic nomogram (Figure 4C). Results showed that when the threshold probability was more than 5% in the training cohort and 2% in the validation cohort, using the nomogram provided more benefit than either assuming that all BI-RADS 4 lesions were malignant or assuming that all BI-RADS 4 lesions were benign.

**Figure 5:**
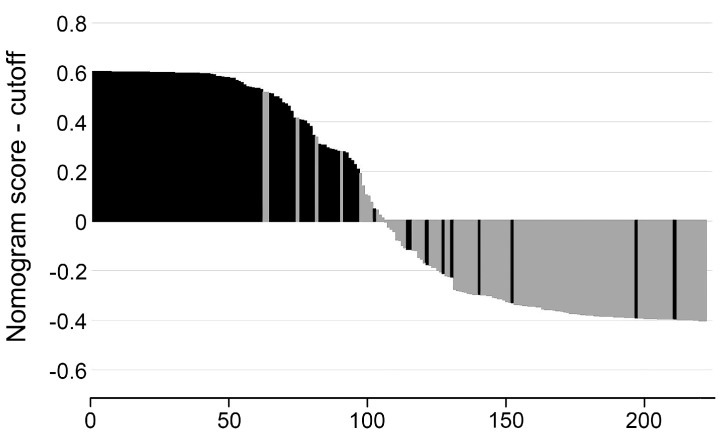
Bar diagrams of BI-RADS 4 lesions according to the optimal cutoff value of nomogram score. Vertical axis refers to the nomogram score minus the optimal cut-off value (i.e., Nomo-score −0.40). Up and down bars refer to the biopsy group (Nomo-score > 0.4, *n* = 106) the follow-up group (Nomo-score ≤ 0.4; *n* = 117), respectively. Black and grey bars refer to actual malignant and benign lesions, respectively.

To evaluate the value of the ABVS radiomic nomogram in reducing unnecessary biopsy of BI-RADS 4 lesions. We divided all BI-RADS 4 lesions into a follow-up group (Nomo-score ≤ 0.4; *n* = 117) and a biopsy group (Nomo-score > 0.4, *n* = 106), and plotted a Nomo-score bar diagram (Figure 5). If biopsies were only performed in the biopsy group, the unnecessary biopsy rate would be reduced from 53. 8% (120/223) to 11.3% (12/106), but 4.0% (9/223) of malignant BI-RADS 4 lesions were unfortunately missed. Among the nine missed malignant BI-RADS 4 lesions, 77.8% (7/9) were BI-RADS 4A lesions, and 22.2% (2/9) were BI-RADS 4B lesions. Except for one BI-RADS 4A lesion (complex cystic and solid) with a size of 54 mm, the size of other eight lesions ranged from 5 to 16 mm (mean size, 9 mm), and six of them were smaller than 10 mm. Histopathology confirmed that seven of the nine missed malignant BI-RADS 4 lesions were invasive ductal carcinoma and two were ductal carcinoma in situ.

## 4. Discussion

In this study, we first developed and validated a radiomic nomogram that combines ABVS radiomic score, lesion size and BI-RADS 4 subcategories for noninvasive, individualized distinction benign and malignant BI-RADS 4 lesions. The radiomic nomogram demonstrated favorable discrimination in both the training and the validation cohorts as well as good calibration and usefulness. Moreover, 42.5% of unnecessary biopsy of BI-RADS 4 lesions would be reduced by using the optimal cutoff value (0.4) of the ABVS radiomic nomogram.

To date, only few studies have investigated a predictive model for breast cancer with radiomic in BI-RADS 4 lesions, and no studies based on ABVS. Luo et al. [16] reported that using radiomic nomogram based on conventional US for predicting breast malignancy in BI-RADS 4 or 5 lesions, and yield an AUC of 0.928 in the validation group, which is comparable to our ABVS radiomic nomogram (0.928 vs. 0.925). Although the performance only in BI-RADS 4 lesions has not been reported by Luo et al., we speculate that our ABVS radiomic nomogram may superior to them in distinguishing benign and malignant BI-RADS 4 lesions. Since BI-RADS 5 lesions have typical signs of malignancy, and almost all lesions are malignant (>95% risk of malignancy), the inclusion of BI-RADS 5 lesions may improve the prediction performance of the radiomic nomogram to some extent. Similarly, Hu et al. [15] developed and validated an MRI radiomic nomogram for differentiating benign and malignant BI-RADS 4 lesions with an AUC of 0.79 in the testing set, which was significantly lower than that of our ABVS radiomic nomogram (AUC, 0.925), and the possible reasons are as follow. First, we included a larger sample size of BI-RADS 4 lesions (223 vs. 86), which may be the main reason. Since larger datasets could provide larger training samples, thus better accuracies for radiomic models [26]. Second, Hu et al. only extracted radiomic features from the largest long-axis cross-section image, while we extracted multiplanar (axial, coronal, and sagittal) radiomic features of the lesion, thus better reflecting the biological behavior and tumoral heterogeneity, providing higher performance in predicting malignant BI-RADS 4 lesions. In our study, in addition to radiomic scores, the lesion size and BI-RADS 4 subcategories were also included in the nomogram based on the results of multivariate logistic regression analysis. The AUC of the nomogram was significantly higher than that of the radiomic score in the training and validation cohorts (*p* = 0.002 and *p* = 0.04), which implies that lesion size and BI-RADS 4 subcategories also play important roles in the differential diagnosis of benign and malignant BI-RADS 4 lesions. Therefore, in routine clinical practice, we need to assess the subcategories and size of BI-RADS 4 lesions as accurately as possible, which is consistent with previous studies [27].

**Figure 6:**
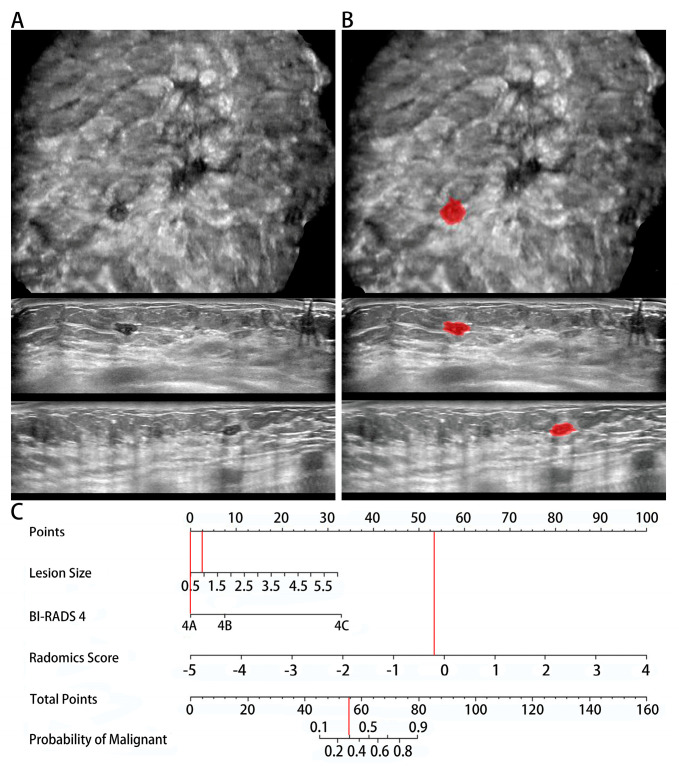
A false negative case of the radiomic nomogram. (**A**) Coronal, axial, and sagittal ABVS images showed a 0.9 cm BI-RADS 4A lesion, which was pathologically confirmed as invasive breast cancer. (**B**) Lesions were manually segmented on axial, sagittal and coronal ABVS images. (**C**) The probability of malignant calculated by the radiomic nomogram was less than 0.4, and the lesion will be missed diagnosis.

According to the latest edition of the ACR BI-RADS-US lexicon, biopsy is recommended for lesions classified as BI-RADS 4. In our study, 53.8% (120/223) of BI-RADS 4 lesions were pathologically confirmed to be benign, meaning that more than half of BI-RADS 4 lesions received unnecessary biopsies. Recently, as a supplement to conventional US, elastography and contrast-enhanced ultrasound (CEUS) have provided more diagnostic information for BI-RADS 4 lesions, and 32.6–44.3% of unnecessary biopsies could be eliminated by these supplement methods [28,29]. However, elastography has technical limitations such as artefacts and lack of reproducibility, while CEUS is more expensive and requires intravenous injection of contrast agents. Moreover, both elastography and CEUS require complex operations and considerable operator expertise [28]. In our study, we used the ABVS radiomic nomogram, which is a convenient, objective and low-cost method to achieve a similar performance (42.5%) in reducing unnecessary biopsies for BI-RADS 4 lesions. However, special care should be taken when using the ABVS radiomic nomogram in small BI-RADS 4A lesions. In our study, 4.0% (9/223) of malignant BI-RADS 4 lesions were unfortunately missed, of which 4A lesions (7/9, 77.8%) and small-sized (<10 mm) lesions (6/9, 66.7%) accounted for the majority. The possible reasons for missed diagnosis are as follows. First, integrating the lesion size and BI-RADS 4 subcategories into the ABVS radiomic nomogram indeed improves the identification performance of the nomogram, but small 4A lesions are also easily identified as benign due to their low score (Figure 6). Secondly, the boundaries of some characteristics between benign and malignant 4A lesions are indistinct or even inverted, especially small size lesions [30]. In addition, the small proportion (12/223, 5.4%) of malignant 4A lesions in this study may also impair the performance of ABVS radiomic nomogram in these lesions. Therefore, the ABVS radiomic nomogram may be a potential tool to reduce unnecessary biopsies of BI-RADS 4 lesions, but its ability in small BI-RADS 4A lesions needs to be improved. Additionally, the performance of the ABVS radiomic nomogram in some rare malignant breast tumors (such as fibromatosis-like spindle cell carcinoma, myofibroblastic sarcoma, etc.) is unknown [31], since all malignant BI-RADS 4 lesions are invasive cancers in our study. Future research should include more types of malignant lesions to promote the clinical application of this method.

In recent years, ABVS has received increasing attention from researchers because it overcomes some of the major limitations of conventional US and has good performance in both the screening setting and the diagnostic setting of breast cancer [18,32]. However, some inherent characteristics of ABVS may affect its promotion and application in the clinic. For one patient, approximately 2000 images were typically generated by ABVS examination, and the sheer volume of images poses a great challenge for radiologists to interpret these images [18]. A study showed that the mean interpretation time of ABVS examination for one patient is approximately 9 min, and the time will increase significantly when abnormal or malignant lesions are detected [33]. Additionally, the detection and classification of breast lesions by ABVS depends on the experience of radiologists [34], and limited experience will reduce diagnostic sensitivity and specificity of ABVS in breast cancer [35]. However, in our research, the ABVS radiomic nomogram can distinguish benign and malignant BI-RADS 4 lesions without dependence on the experience of radiologists, and only three ABVS images of the lesion are required, which may simplify the diagnostic workflow and extend the clinical application of ABVS to the treatment and prognosis of breast cancer [36], since ABVS-based radiomics may also be useful in predicting the molecular subtypes of breast cancer, axillary lymph node metastasis, or response to neoadjuvant therapy.

There are still several limitations in this study. First, the small sample size from a single center and the lack of an external validation set may limit the robustness and reproducibility of the radiomic nomogram. Therefore, multicenter studies with considerably large datasets are needed in future research. Second, although we extract radiomic features from the axial, sagittal, and coronal ABVS images of each lesion, the three-dimensional radiomic features may contain more tumor information to identify the benign and malignant lesions, which is our future research direction. Third, manually segmentation may have inter-operator variability, and semiautomatic or automatic segmentation methods should be combined in further research.

## 5. Conclusions

In conclusion, the ABVS radiomic nomogram showed satisfactory discrimination performance between benign and malignant BI-RADS 4 lesions as well as good calibration. It may be a potential tool to reduce unnecessary biopsies of BI-RADS 4 lesions, but its ability in small BI-RADS 4A lesion identification needs to be improved. Large-scale multicenter studies are warranted to further promote the performance of ABVS radiomic nomogram.

## Figures and Tables

**Table 1:** Clinical basic characteristics and ultrasound features in training and validation cohorts.

Characteristics	Training Cohort (*n* = 178)	Validation Cohort (*n* = 45)	Statistic (χ^2^ or t)	*p*
Age(year)	48.9 ± 12.3	51.3 ± 12.1	−1.206	0.229
BI-RADS 4 category				
4a	84 (47.2%)	20 (44.4%)	1.044	0.593
4b	36 (20.2%)	7 (15.6%)		
4c	58 (32.6%)	18 (40.0%)		
Breast density				
A (<25% dense tissue)	15 (8.4%)	3 (6.7%)	0.680	0.878
B (25–50% dense tissue)	64 (36.0%)	17 (37.8%)		
C (51–75% dense tissue)	68 (38.2%)	19 (42.2%)		
D (>75% dense tissue)	31 (17.4%)	6 (13.3%)		
Menopausal				
Pre-menopausal	106 (59.6%)	19 (42.2%)	3.703	0.054
postmenopausal	72 (40.4%)	26 (57.8%)		
Oral contraceptives				
Yes	22 (12.4%)	9 (20.0%)	1.752	0.186
No	156 (87.6%)	36 (80.0%)		
Family history of breast cancer				
Yes	16 (9.0%)	8 (17.8%)	2.046	0.153
No	162 (91.0%)	37 (82.2%)		
Smoking history				
Yes	7 (3.9%)	5 (11.1%)	2.362	0.124
No	171 (96.1%)	40 (88.9%)		
Alcohol drinking history				
Yes	7 (3.9%)	4 (8.9%)	0.973	0.324
No	171 (96.1%)	41 (91.1%)		
Location of lesions				
Left	103 (57.9%)	26 (57.8%)	0.000	0.992
Right	75 (42.1%)	19 (42.2%)		
Lesion size (cm)	1.9 ± 1.0	2.0 ± 1.1	−0.600	0.549
Shape				
Regular	32 (18.0%)	13 (28.9%)	2.655	0.103
Irregular	146 (82.0%)	32 (71.1%)		
Orientation				
Parallel	105 (59.0%)	27 (60.0%)	0.015	0.902
Not parallel	73 (41.0%)	18 (40.0%)		
Margin				
Circumscribed	19 (10.7%)	9 (20.0%)	2.845	0.092
Not circumscribed	159 (89.3%)	36 (80.0%)		
Posterior echo				
No posterior echo	69 (38.8%)	21 (46.7%)	1.163	0.762
Enhancement	31 (17.4%)	8 (17.8%)		
Shadowing	52 (29.2%)	11 (24.4%)		
Combined pattern	26 (14.6%)	5 (11.1%)		
Echo pattern *				
Complex cystic and solid	14 (7.9%)	4 (8.9%)	0.051	0.822
Hypoechoic	164 (92.1%)	41 (91.1%)		
Calcification				
Yes	88 (49.4%)	28 (62.2%)	2.352	0.125
No	90 (50.6%)	17 (37.8%)		
Radiomic score	0.185 ± 1.659	0.257 ± 1.689	0.255	0.799

BI-RADS = breast imaging reporting and data system; Lesion size was defined as the maximum diameter on ABVS images. * The breast lesions in this study were only hypoechoic echo pattern and complex cystic and solid echo pattern. The differences in characteristic variables (age, lesion size, radiomic score) between the two cohorts were compared by two-sample *t*-test, whereas Chi-square tests was conducted to other variables. *p* < 0.05.

**Table 2:** Results of the univariate and multivariate logistic regression analyses based on the training group.

Variables	Univariate Logistic Regression		Multivariate Logistic Regression	
	OR (95% CI)	*p*-Value	OR (95% CI)	*p*-Value
Age (years)	1.085 (1.053, 1.118)	<0.001	1.060 (0.965, 1.165)	0.226
BI-RADS 4 category				
4A	Ref.		Ref.	
4B	0.955 (0.459, 1.989)	<0.001	NA	NA
4C	4.978 (1.656, 5.963)	<0.001	4.794 (0.803, 8.624)	<0.001
Breast density				
A (<25% dense tissue)	Ref.		Ref.	
B (25–50% dense tissue)	2.283 (1.231, 4.234)	0.009	1.741 (0.460, 6.584)	0.414
C (51–75% dense tissue)	0.648 (0.355, 1.184)	0.158	NA	NA
D (>75% dense tissue)	0.234 (0.091, 0.602)	0.003	0.526 (0.095, 2.902)	0.461
Menopausal (Pre-/post-menopausal)	0.244 (0.130, 0.456)	<0.001	1.004 (0.143, 7.072)	0.997
Oral contraceptives (Yes/No)	1.227 (0.440, 3.423)	0.696	NA	NA
Family history of breast cancer (Yes/No)	1.226 (0.439, 3.423)	0.696	NA	NA
Smoking history (Yes/No)	1.637 (0.356, 7.532)	0.527	NA	NA
Alcohol drinking history (Yes/No)	3.141 (0.593, 16.629)	0.178	NA	NA
Location of lesion (Left/Right)	0.729 (0.402, 1.320)	0.296	NA	NA
Lesion size (cm)	2.369 (1.636, 3.431)	<0.001	2.838 (1.468, 5.486)	0.002
Shape (Regular/ Irregular)	1.785 (0.771, 4.133)	0.176	NA	NA
Orientation (Parallel/ Not parallel)	0.458 (0.252, 0.8340)	0.011	0.448 (0.134, 1.499)	0.193
Margin (Circumscribed/Not circumscribed)	0.445 (0.167, 1.189)	0.106	NA	NA
Posterior echo				
Enhancement	Ref.		Ref.	
No posterior echo	0.815 (0.447, 1.483)	0.502	NA	NA
Shadowing	1.173 (0.620, 2.218)	0.624	NA	NA
Combined pattern	0.368 (0.147, 0.921)	0.033	NA	NA
Echo pattern				
Hypoechoic	Ref.		Ref.	
Complex cystic and solid	1.224 (0.411, 3.642)	0.717	NA	NA
Calcification (Yes/No)	0.780 (0.435, 1.397)	0.403	NA	NA
Radiomic score	0.269 (0.186, 0.341)	<0.001	0.206 (0.104, 0.407)	<0.001

Lesion size was defined as the maximum diameter on ABVS images. Ref = reference; NA = values were not available; CI = confidence interval.

**Table 3:** Performance of the radiomic nomogram in training cohort, validation cohort and combined cohort.

	Training Cohort (*n* = 178)	Validation Cohort (*n* = 45)	Combined Cohort (*n* = 223)
AUC (95% CI)	0.96 (0.93 to 0.98)	0.92 (0.82 to 0.97)	0.95 (0.92 to 0.98)
Accuracy (%)	91.6%	86.7%	90.6%
Sensitivity (%)	90.2%	83.3%	88.7%
Specificity (%)	92.7%	90.5%	92.3%
PPV (%)	91.4%	90.9%	91.3%
NPV (%)	91.8%	82.6%	90.0%

AUC = area under the curve; CI = confidence interval; PPV = positive predictive value; NPV = negative predictive value.

## Data Availability

The data presented in this study are available upon reasonable request from the corresponding author.

## Supplementary Materials

The following supporting information can be downloaded at: https://www.mdpi.com/article/10.3390/diagnostics12010172/s1, S1: The detailed packages of R software in this study, S2: Formulas of the radiomics score in this study, Table S1: Radiomics feature used in the study, Table S2: Clinical basic characteristics and ultrasound features in training and validation cohorts for benign and malignant lesions.
