# Supplementary material for: Automated Breast Volume Scanner (ABVS)-Based Radiomic Nomogram: A Potential Tool for Reducing Unnecessary Biopsies of BI-RADS 4 Lesions
Source: Diagnostics (Basel). 2022 Jan 12;12(1):172. doi: 10.3390/diagnostics12010172 (PMC8774686; doi:10.3390/diagnostics12010172)
Supplement: Supplementary file 1 [file diagnostics-12-00172-s001.zip › diagnostics-1461705-supplementary.pdf]

Supplementary Material

Supplemental S1. The detailed packages of R software in this study.

The "mRMRe" and "glmnet" packages were used to perform the mRMR algorithm and LASSO logistic regression. The "rms" package was used for radiomic score construction. Univariate and multivariate logistic regression analyses used the "glm" package. The "rms" package was used for nomogram construction and calibration plotting. ROC curves were plotted using "pROC" package. The "rmda" package was used to perform the decision curve.

Supplemental S2. Formulas of the radiomics score in this study.

Radiomics score = 0.203294524 + 0.115755071* C_GeoFh - 0.544859545* C_GeoNsz + 0.356015436* C_S04DifEntrp + 0.96599925* C_Area_S55_A - 0.009999382* C_Horzl_ShrtREmp - 0.062574886* C_GrNonZeros - 0.668516041* T_GeoNsz + 0.218110427* T_S11InvDfMom - 0.017262842* T_S02Correlat - 0.184686195* T_S22Correlat_A + 0.014801496* T_Area_S55 - 0.132782005* T_S55DifEntrp - 0.339411174* S_GeoNc - 0.69294567* S_GeoNsz - 0.09479637* S_S11Correlat + 0.091536746* S_S11InvDfMom_A + 0.126878376* S_S22Contrast + 0.198291556* S_S55Contrast_A - 0.006180319* S_45dgr_Fraction

Table S1. Radiomics feature used in the study.

Radiomics features	Description
Histogram	Mean, variance, skewness, kurtosis, and 1st, 10th, 50th, 90th, and 99th percentiles
Geometry	Descriptors of the two-dimensional size and shape of the ROI
Absolute gradient	Mean, variance, skewness, kurtosis, and percentage of pixels with nonzero gradient
GLCM	Angular second moment, contrast, correlation, sum of squares, inverse difference moment, sum average, sum variance, sum entropy, entropy, difference variance, and difference entropy; parameters are computed up to 20 times for (*d*, 0), (0, *d*), (*d*, *d*), (*d*, -*d*) and the *d* can take values of 1, 2, 3, 4 and 5
RLM	Run-length nonuniformity, gray-level nonuniformity, long-run emphasis, short-run emphasis, and fraction of image in runs; parameters are computed 4 times for horizontal, 45°, vertical, and 135° directions
AM	Assumes a local interaction between image pixels in that pixel intensity is a weighted sum of neighborhood pixel intensities and has 5 unknown model parameters - the standard deviation of the driving noise e_s and the model parameter vector θ = [θ1, θ2, θ3, θ4]
Wavelet transform	The discrete wavelet transform is a linear transformation that operates on a data vector whose length is an integer power of two, transforming it into a numerically different vector of the same length

e_s denotes an independent and identically distributed noise; θ is a vector of model parameters; ROI = region of interest; GLCM = gray-level co-occurrence matrix; RLM = run-length matrix; AM = autoregressive model.

Table S2. Clinical basic characteristics and ultrasound features in training and validation cohorts for benign and malignant lesions

Characteristics	Training cohort (n=178)			Validation cohort (n=45)		
	**Malignant (n=81)**	**Benign (n=97)**	***p***	**Malignant (n=22)**	**Benign (n=23)**	***p***
Age(year)	54.5±12.5	44.2±9.8	<0.001	58.1±9.0	45.1±12.4	0.001
BI-RADS 4 category						
4a	11(13.6%)	73(75.3%)	<0.001	1(4.5%)	19(82.6%)	<0.001
4b	16(19.8 %)	20(20.6%)		3(13.6%)	4(17.4%)	
4c	54(66.7%)	4(4.1%)		18(81.8%)	0(0.0%)	
Breast density						
A	11(13.6%)	4(4.1%)	<0.001	3(13.6%)	0(0.0%)	0.308
B	37(45.7%)	27(27.8%)		9(40.9%)	8(34.8%)	
C	27(33.3%)	41(42.3%)		7(31.8%)	12(52.2%)	
D	6(7.4%)	25(25.8%)		3(13.6%)	3(13.0%)	
Menopausal						
Pre-menopausal	33(40.7%)	73(75.3%)	<0.001	5(22.7%)	14(60.9%)	0.004
postmenopausal	48(59.3%)	24(24.7%)		17(77.3%)	9(39.1%)	
Oral contraceptives						
Yes	10(12.3%)	12(12.4%)	0.696	2(9.1%)	7(30.4%)	0.236
No	71(87.7%)	85(87.6%)		20(90.9%)	16(69.6%)	
Family history of breast cancer						
Yes	8(9.9%)	8(8.2%)	0.696	4(18.2%)	4(17.4%)	0.465
No	73(90.1%)	89(91.8%)		18(81.8%)	19(82.6%)	
Smoking history						
Yes	4(4.9%)	3(3.1%)	0.523	2(9.1%)	3(13.0%)	0.633
No	77(95.1%)	94(96.9%)		20(90.9%)	20(87.0%)	
Alcohol drinking history						
Yes	5(6.2%)	2(2.1%)	0.158	2(9.1%)	2(8.7%)	1.000
No	76(93.8%)	95(97.9%)		20(90.9%)	21(91.3%)	
Location of lesions						
Left	51(63.0%)	52(53.6%)	0.296	11(50.0%)	15(65.2%)	0.525
Right	30(37.0%)	45(46.4%)		11(50.0%)	8(34.8%)	
Lesion size (cm)	2.3±0.9	1.6±0.9	<0.001	2.5±1.2	1.6±0.8	0.010
Shape						
Regular	7(8.6%)	19(19.6%)	0.042	3(13.6%)	10(43.5%)	0.018
Irregular	74(91.4%)	78 (80.4%)		19(86.4%)	13(56.5%)	

Orientation						
Parallel	48(59.3%)	57(58.8%)	0.020	8(36.4%)	19(82.6%)	<0.001
Not parallel	33(40.7%)	40(41.2%)		14(63.6%)	4(17.4%)	
Margin						
Circumscribed	8(9.9%)	11(11.3%)	0.764	2(9.1%)	7(30.4%)	0.130
Not circumscribed	73(90.1%)	86(88.7%)		20(90.9%)	16(69.6%)	
Posterior echo						
No posterior echo	30(37.0%)	39(40.2%)	0.625	10(45.5%)	11(47.8%)	0.256
Enhancement	17(21.0%)	14(14.4%)		2(9.1%)	6(26.1%)	
Shadowing	21(25.9%)	31(32.0%)		7(31.8%)	4(17.4%)	
Combined pattern	13(16.0%)	13(13.4%)		3(13.6%)	2(8.7%)	
Echo pattern*						
Complex cystic and solid	5(6.2%)	9(9.3%)	0.451	0(0.0%)	4(17.4%)	0.114
Hypoechoic	76(93.8%)	88(90.7%)		22(100.0%)	19(82.6%)	
Calcification						
Yes	52(64.2%)	36(37.1%)	<0.001	15(68.2%)	13(56.5%)	0.507
No	29(35.8%)	61(62.9%)		7(31.8%)	10(43.5%)	
Radiomic score	-0.944±1.497	1.145±1.085	<0.001	-0.838±1.740	1.244±0.845	<0.001

BI-RADS = breast imaging reporting and data system; Rad-score = radiomics score; lesion size was defined as the maximum diameter on ABVS images.

* The breast lesions in this study are only hypoechoic echo pattern and complex cystic and solid echo pattern.

The differences in characteristic variables (age, lesion size, radiomic score) between the two cohorts were compared by two-sample t test, whereas Chi-square tests was conducted to other variables. $P < 0.05$
